# The FurA regulon in *Anabaena* sp. PCC 7120: *in silico* prediction and experimental validation of novel target genes

**DOI:** 10.1093/nar/gku123

**Published:** 2014-02-06

**Authors:** Andrés González, Vladimir Espinosa Angarica, Javier Sancho, María F. Fillat

**Affiliations:** ^1^Departamento de Bioquímica y Biología Molecular y Celular, Universidad de Zaragoza, 50009 Zaragoza, Spain, ^2^Instituto de Biocomputación y Física de Sistemas Complejos (BIFI), Universidad de Zaragoza, 50018 Zaragoza, Spain and ^3^Unidad Asociada BIFI-IQFR (CSIC), 28006 Madrid, Spain

## Abstract

In the filamentous cyanobacterium *Anabaena* sp. PCC 7120, the ferric uptake regulator FurA functions as a global transcriptional regulator. Despite several analyses have focused on elucidating the FurA-regulatory network, the number of target genes described for this essential transcription factor is limited to a handful of examples. In this article, we combine an *in silico* genome-wide predictive approach with experimental determinations to better define the FurA regulon. Predicted FurA-binding sites were identified upstream of 215 genes belonging to diverse functional categories including iron homeostasis, photosynthesis and respiration, heterocyst differentiation, oxidative stress defence and light-dependent signal transduction mechanisms, among others. The probabilistic model proved to be effective at discerning FurA boxes from non-cognate sequences, while subsequent electrophoretic mobility shift assay experiments confirmed the *in vitro* specific binding of FurA to at least 20 selected predicted targets. Gene-expression analyses further supported the dual role of FurA as transcriptional modulator that can act both as repressor and as activator. In either role, the *in vitro* affinity of the protein to its target sequences is strongly dependent on metal co-regulator and reducing conditions, suggesting that FurA couples *in vivo* iron homeostasis and the response to oxidative stress to major physiological processes in cyanobacteria.

## INTRODUCTION

Cyanobacteria are oxygen-evolving photosynthetic prokaryotes found in virtually all ecosystem habitats on the Earth (1). As primary producers, these organisms are major participants in global carbon cycles, playing key roles in both food-chain dynamics and biogeochemistry of oceans and freshwaters (2). In addition, many cyanobacteria are capable of fixing atmospheric dinitrogen in the absence of combined nitrogen sources, providing the largest fraction of total nitrogen fixation in the open ocean (3). Since oxygenic photosynthesis and nitrogen fixation are incompatible processes because nitrogenase is inactivated by oxygen, some diazotrophic cyanobacteria spatially separate these activities through multi-cellularity and cellular differentiation. In those species, the absence of combined nitrogen triggers a complex developmental process of differentiation resulting in highly specialized cells known as heterocysts, which provide the appropriate micro-oxic environment for the expression and function of nitrogenase (4,5).

Several large-scale studies have indicated that primary production and nitrogen fixation in the open ocean is greatly influenced by iron bioavailability (6,7), thereby implying that iron may be an important limiting factor for cyanobacterial growth in nature. Like almost all life forms, cyanobacteria have an absolute dependence of iron for growth and optimal development of their major physiological processes, particularly photosynthesis and nitrogen fixation. Iron serves as cofactor for every membrane-bound protein complex and other mobile electron carriers within the photosynthetic apparatus (8), which determines an iron quota ∼10 times higher than that exhibited by a similarly sized non-photosynthetic bacterium (9). Additionally, diazotrophic cyanobacteria have significant further iron requirements compared with other phototrophs, due to the abundance of iron-containing enzymes in the nitrogen-fixation machinery (10).

Because iron is scarcely soluble in aqueous environments at neutral pH, cyanobacteria have evolved strategies to efficiently scavenge (11,12), to incorporate (13,14), and to store this essential micronutrient in the cell (9,15). However, an excess of free intracellular iron is extremely deleterious because of it catalyzes the formation of reactive oxygen species through Fenton reactions, leading to oxidative stress (16). Iron metabolism is, therefore, tightly regulated in order to maintain the intracellular concentration within non-toxic levels.

For most gram-negative and several gram-positive bacteria, the effective balance between iron acquisition and protection against oxidative stress is controlled by a global transcriptional regulator known as Fur, which stands for ferric uptake regulator (17). Fur typically acts as a transcriptional repressor, which sense intracellular free iron and modulates transcription in response to iron availability. This is accomplished by binding Fur–Fe^2+^ complexes to *cis*-acting regulatory elements known as Fur boxes, located in the promoter regions of iron-responsive genes (18). Under iron-restricted conditions, the metal co-repressor is released and the repressor becomes inactive, allowing the transcription of target genes. More recently, Fur-mediated direct and indirect activation of transcription involving a variety of mechanisms have been established (19–21). As a global regulator, Fur not only controls the expression of iron acquisition and storage systems, but also a plethora of genes and operons belonging to a broad range of functional categories (21–24). All these genes constitute the Fur regulon.

In the filamentous, nitrogen-fixing, heterocyst-forming cyanobacterium *Anabaena* sp. PCC 7120, the protein FurA is the master regulator of iron homeostasis (25). FurA is a constitutive and essential protein (26,27), whose expression increases under iron deprivation (28), oxidative stress (29) and during heterocyst differentiation (4,30). Our previous *in vitro* and *in vivo* analyses have shown that FurA plays a global regulatory role (25,31–34), modulating the expression of a variety of genes involved in different physiological processes including iron metabolism, photosynthesis, heterocyst differentiation, oxidative stress defences and cellular morphology, among others. However, only a few examples of *cis–trans* regulatory links for this regulator are known (35), and there is still a lack of knowledge of the Fur regulon from a global point of view. In the present article, we combined a bioinformatics predictive methodology with experimental analyses to get a more complete picture of the FurA regulon in *Anabaena* sp. PCC 7120. The novel Fur targets identified go beyond those involved in iron homeostasis in heterotrophic bacteria. We have identified FurA boxes associated with a variety of enzymes, transporters and regulators, many of them included in gene clusters and putative operons which greatly expands the scope of Fur-regulated genes and provides new insights into the role of Fur proteins in cyanobacteria.

## MATERIALS AND METHODS

### Weight-matrix-based FurA-binding sites prediction model

A set of eight FurA-binding sequences experimentally identified in previous studies by means of DNase I-footprinting assays (25,36) was used as training set for the construction of a weight-matrix using the CONSENSUS algorithm (37). The algorithm was set to freely pick out the number of significant sequences to be used in the generation of matrices with different widths. From the knowledge of Fur protein matrices in other organisms (38,39), we also instructed the algorithm to construct non-palindromic matrices of widths between 16 and 22 bases. The most statistically significant matrix was selected according to its expected frequency and information content. Based on the scores obtained for the sites in the training set, we selected for this matrix a cut-off value of 10 bits to be used in the predictive stage. To ensure the suitability of our *in silico* model for performing a genome-wide scanning, a benchmarking test was set up in order to assess the reliability of the matrix to discriminate between cognate and non-cognate binding sites. As non-cognate binding sites, we considered the genomic sequences between two consecutive convergent genes (40). Hence, we extracted all the stretches between consecutive convergent genes into the complete *Anabaena* sp. PCC 7120 genome downloaded from the CyanoBase (41), and scanned them with the FurA matrix.

### Bacterial strains and growth conditions

Wild-type *Anabaena* sp. PCC 7120 and its derivative *furA*-overexpressing strain AG2770FurA (32) were routinely grown photoautotrophically in BG-11 medium (42) at 30°C, supplemented with neomycin 50 µg/ml in the case of strain AG2770FurA. Cyanobacterial strains were cultured in 250 ml Erlenmeyer flasks containing 60 ml culture medium under continuous white light illumination (20 μE/m^2^s) and shaking (120 rpm). Chlorophyll *a* (Chl) was determined in methanol extracts (13).

For iron depletion experiments, exponentially growing filaments (3–5 µg Chl/ml) from standard BG-11 were collected by filtration, washed three times with BG-11 medium without iron (BG-11_-Fe_), re-suspended in the same iron-free medium and further grown for 72 h. Glassware used in these experiments was soaked with 6 M HCl and extensively rinsed with Milli-Q water to remove residual iron. For transcriptional analyses during heterocyst differentiation, the *Anabaena* sp. strains were cultured as previous described (34). Exponentially growing filaments from standard BG-11 were collected by filtration, washed twice with BG-11 medium without NaNO_3_ (BG-11_0_) and resuspended in the same nitrogen-free medium. Cultures were further incubated until mature heterocysts were observed (24 h under our experimental conditions).

### Electrophoretic mobility shift assays

Recombinant *Anabaena* sp. PCC 7120 FurA protein was expressed in *Escherichia coli* BL21 (DE3) and purified according to previously described methods (43). The promoter regions of each gene of interest were obtained by PCR using the primers described in Supplementary Table S1. Electrophoretic mobility shift assays (EMSA) were performed as described previously (36). Briefly, 120–140 ng of each DNA fragment were mixed with recombinant FurA protein at concentrations of 300, 500 and 700 nM in a 20-µl reaction volume containing 10 mM bis–Tris (pH 7.5), 40 mM potassium chloride, 100 µg/ml bovine serum albumin, 1 mM DTT, 100 µM manganese chloride and 5% glycerol. In some experiments, EDTA was added to a final concentration of 200 μM. To ensure the specificity of EMSA, the promoter region of *Anabaena* sp. *nifJ* (*alr1911*) gene was included as non-specific competitor DNA in all assays (31). Mixtures were incubated at room temperature for 20 min, and subsequently separated on 4% non-denaturing polyacrylamide gels in running buffer (25 mM Tris, 190 mM glycine) at 90 V. Gels were stained with SYBR® Safe DNA gel stain (Invitrogen) and processed with a Gel Doc 2000 Image Analyzer (Bio-Rad).

### Semi-quantitative reverse transcription-PCR

Total RNA from *Anabaena* sp. strains was isolated as described previously (44). Samples of 1 µg RNA were heated at 85°C for 10 min and used as templates for the first-strand cDNA synthesis. Residual DNA in RNA preparations was eliminated by digestion with RNase-free DNase I (Roche). The absence of DNA was checked by PCR. Reverse transcription was carried out using SuperScript retrotranscriptase (Invitrogen) in a 20-µl reaction volume containing 150 ng of random primers (Invitrogen), 1 mM dNTP mix (GE Healthcare) and 10 mM DTT. The sequences of the specific primers used for semi-quantitative reverse transcription-PCR (sqRT-PCR) reactions are defined in Supplementary Table S1. Housekeeping gene *rnpB* (45) was used as a control to compensate for variations in the input of RNA amounts and normalize the results. Exponential phase of PCR for each gene was determined by measuring the amount of PCR product at different time intervals. For the final results, 20–23 cycles at the early exponential phase were used in all genes analyzed. The PCR products were resolved by electrophoresis in 1% agarose gels, stained with ethidium bromide and analyzed using a Gel Doc 2000 Image Analyzer (Bio-Rad).

Relative induction ratio for each gene was calculated as the average of ratios between signals observed in two environmental conditions of interest in three independent determinations. Signal assigned to each gene corresponded to the intensity of its DNA band in the agarose gel stained with ethidium bromide normalized to the signal observed for the housekeeping gene *rnpB* in each strain*.*

## RESULTS

### Reliability of the *in silico* model

In order to characterize the FurA regulon in the nitrogen-fixing cyanobacterium *Anabaena* sp. PCC 7120, we carried out a genome-wide search for putative binding sites using a weight-matrix-based *in silico* approach. The FurA recognition weight-matrix was derived from a set of previously validated binding sites determined by DNase I footprinting assays (25,36). After building the model, we were interested in testing its suitability to perform a genome-wide scanning, thus we set up a benchmarking test to assess the reliability of the matrix to discern cognate and non-cognate binding sites. The selection of non-cognate binding sites—i.e. a group of sequences not bound by a given transcriptional factor*—*is a complex issue because it is impossible to know in advance the genes that are not modulated by a global regulator like Fur in the genome. Since there are different methodological propositions to overcome those limitations (40), we have used as negative set all the *Anabaena* genomic sequences between two consecutive convergent genes, in which the frequency of binding sequences for any transcription factor is expected to be low. When scanning these genomic regions, the score distribution showed a mean value around −11 bits (Supplementary Figure S1), which means that most of non-cognate sequences resulted in negative scores. The score range of a weight-matrix is defined as the values between the minimum and the maximum scores that can be obtained for the sequence more dissimilar to the model and the consensus sequence, respectively. The matrix used in our study had a score range between −41.7 and 17.2 bits. The score distribution of the non-cognate sequences indicated that most of them obtained negative scores, which constitutes the expected behaviour for a matrix with a good classification performance. In contrast, the sites recognized by the transcriptional regulator in experimental determinations scored at higher values, close to the matrix maximum score. The statistical assessment of the performance of our model showed that the matrix was effective at discriminating between cognate and non-cognate sites since for the selected cut-off score of 10 bits, the parametric ℘-value for distribution comparison is rather low (7.43 × 10^−^^5^), with a significance value of 0.01% (Supplementary Figure S1).

### Predicted FurA-binding sites and associated genes in the *Anabaena* sp. PCC 7120 genome

Given the high performance of the matrix built, we scanned the complete genome of *Anabaena* sp. PCC 7120, including the chromosome and all the six natural cytosolic plasmids (46), to locate putative FurA-binding sites. The complete list of predicted FurA-binding sites and their associated genes is shown in Supplementary Table S2. This dataset contained a total of 227 predicted binding sites located in the promoter regions of 200 chromosomal protein-coding genes and 15 additional genes carried in plasmids α, β, γ and δ. Most of these putative FurA targets contained one predicted Fur-binding site; however, 12 genes contained two candidate binding sites into their promoter regions (Supplementary Table S2).

Predicted FurA-binding sites were identified upstream of 101 genes with known or putative functions according to the cyanobacteria genome database CyanoBase (41). Several of these predicted targets are summarized in Table 1. Consistent with the definition of a global transcriptional regulator, putative FurA-binding sites were found into the promoter regions of genes involved in a variety of cellular processes such as photosynthesis, respiration, heterocyst differentiation, oxidative stress defences, energy metabolism, transport across the cell membranes, biosynthesis of different molecules, as well as several regulatory functions, among others.

**Table 1.** A partial list of genes associated with predicted FurA boxes in *Anabaena* sp. PCC 7120 genome

ORF (gene)^a^	Protein description^a^	Location^b^	Predicted FurA box sequence	Distance to ATG	Score
Transport and binding proteins					
Iron acquisition systems					
***alr0397*(*schT*)**	TonB-dependent schizokinen transporter	C	AGATATATTTTAATAAAAT	57	11.29
***alr3242* (*hutA2*)**^c^	TonB-dependent heme transport protein	C	ATTAAAAACCACCAAAAAT	164	10.84
***all1101***^c^	TonB-dependent ferrichrome-iron receptor	C	TGCTAAATCTTGATAATAT	88	10.40
*all2586*^c^	Iron (III) dicitrate ABC transporter permease	C	TACAAAAAGTTGAAAAAAT	217	10.03
*all0473*	Probable Zn^2+^/Fe^2+^ permease	C	ATAAAAATTTATATAAAAT	168	10.01
Other					
*alr4585*	Periplasmic phosphate-binding protein of ABC transporter	C	AATTAAATTCAGAATTAAT	82	13.70
*alr0140*	Periplasmic oligopeptide-binding protein of ABC transporter	C	AATTAAATTTTAATAAAAT	203	12.14
*alr0299*	Periplasmic polyamine-binding protein of ABC transporter	C	AAATAAATATTCTATAAAT	398	11.94
*all0833*(*znuA*)^c^	Periplasmic solute-binding protein of ABC transporter	C	AAAAGTATTCTCAAGATAT	302	11.87
*all4246*	Potassium-dependent ATPase subunit A	C	ATTAAATTTCTGATTAAAT	415	11.32
*alr0190*	ABC transporter, ATP-binding protein	C	CTAAATAATCTCATTTAGT	83	11.30
*alr0990* (*amt4*)^c^	Ammonium transporter	C	ATAAAAATTTTGACCAAAT	82	11.18
*alr1334*	ABC transporter, permease protein	C	TTTTATAGCTTCAACAAAT	147	11.01
*all2113*	Na^+^/H^+^ antiporter	C	ATATAAATTCTGCATATCT	21	10.28
*all2900*	Cation efflux system protein	C	AAATAGATATTCAATTAAC	135	10.22
Regulatory functions					
Transcriptional regulators					
***all1691* (*furA*)**^d^	Ferric uptake regulator	C	ATAAATATTCTCAATAAGC	90	14.61
			TGTTATATTCTCAATTAAC	42	12.95
*all1651*^c^	Transcriptional regulator	C	AATTAAATTTTCAATAATT	1013	13.43
*alr2595*^c^	Transcriptional regulator	C	ATTTAAATATTTAATAAAT	43	11.36
*all3903*	ArsR family transcriptional regulator	C	AAAAATATACTGAAATTGC	27	11.02
Signal transduction					
*all1804*^d^	Two-component hybrid sensor and regulator	C	AAAAAAATACTCAATATGT	1004	14.29
			AACAAAATTTTCAATTTAT	1258	13.48
*all3564*	Two-component sensor histidine kinase	C	TTATAAATTTACATAAAGT	359	11.73
*alr0072*	Two-component response regulator	C	GATTAAAGTCTGAAAAAAT	170	11.73
*all0743* (*cyaD*)	Adenylate cyclase	C	AATAAAATTTTGCTTTAGC	364	10.69
*all2699* (*aphC*)	Two-component sensor histidine kinase (putative photoreceptor)	C	ATTTAAAATTTAAAAATAT	74	10.66
*all3348*	Two-component response regulator	C	TGATAAATCTTGATATTAT	202	10.60
*alr2411*	Serine/threonine kinase	C	AACAAAATTCAAAAAAAGT	270	10.45
*alr3442*	Two-component hybrid sensor and regulator	C	AGAAGAATTTTGAATTGAT	204	10.37
*alr3165* (*asr*)^c^	Bacteriorhodopsin	C	ATAAATATTTTCATTTCAT	118	10.37
*all1704*	Two-component response regulator	C	TACAAAATTCAAAATAAAT	347	10.35
*alr1308*	Two-component sensor histidine kinase	C	AGATAAAGTCTAAAAATGT	162	10.34
*all4963* (*cyaC*)	Adenylate cyclase carring two-component sensor and regulator domains	C	AAATAAAATTTACTTAAAT	311	10.07
*all7584*	Two-component response regulator	beta	AAAAAAATTCTGTAAAATT	98	10.06
Redox regulation					
*asl7641*	Thioredoxin	beta	AATTATATATCCAATAAAT	123	11.19
Photosynthesis and respiration					
***alr2405* (*isiB*)**^d^	Flavodoxin	C	AAATAAATATTCAATAAGT	231	14.83
			CAAAATAGTCTCAATAAAT	277	13.87
*asr4775* (*psaK*)	Photosystem I subunit PsaK	C	AGAAATATCCTGATTATAT	53	12.74
*all4003*^c^	Photosystem II CP43 protein PsbC homolog	C	ATATAAATAGTCAATAAGT	67	11.63
***all1127***^c^	NADH dehydrogenase	C	TGAAAAATTCTCAAGTAGT	425	11.40
*alr0950* (*coxB*)^c^	Cytochrome *c* oxidase subunit II	C	TGCTAAATTTTCCTTAAAT	200	11.26
*all3410*^c^	NAD(P) transhydrogenase subunit alpha	C	AATAGAATTTGCAATATGT	168	10.58
*alr0869* (*ndhF*)^c^^,^^d^	NADH dehydrogenase subunit 5	C	TTAAAAAAACTTAATAAAT	501	10.41
			TAAAAAAGTTTAAAAAAAC	510	10.13
*all4379*	Peptide-chain-release factor 3	C	TTTTAAATTCTCAATTTTT	509	10.38
Heterocyst differentiation					
*alr2817* (*hetC*)	Heterocyst differentiation protein	C	TATAAAATTTTCCTTAAGT	818	12.37
*alr1728*^d^	Fox gene with unknown function	C	AAATAAAGATACAAAAAAT	205	11.85
			TAATGAATACCCAATAAGT	531	10.07
*all0521* (*patA*)	Heterocyst pattern formation regulator	C	TTTTAAATCTTGATTTAAT	34	11.62
*asr1734*	Heterocyst differentiation negative regulator	C	TAATAAAATCAAAAAAAAT	388	10.46
*asl2301* (*patS*)	Heterocyst-inhibiting signalling peptide	C	AGAAATTTTTTCATAAAGT	796	10.36
Oxidative stress defences					
*all5371*	Similar to alkylhydroperoxidase	C	GACAAAATATTGAATAAAT	411	10.56
*all3895* (*flv3a*)	Flavodiiron protein Flv3	C	ATAAAAATTTACAAATATT	73	10.01
Energy metabolism					
*alr0599*	1-Deoxy-xylulose 5-phosphate synthase	C	AAATAAAATTACAACATAT	419	11.89
*alr4670*	Ribose-phosphate pyrophosphokinase	C	AGTTAAATCCACCAAATAT	60	10.67
*all4861* (*pepC*)	Phosphoenolpyruvate carboxylase	C	AAAAATATACTTCAAAAAT	232	10.24
*all1059*	Sucrose synthase	C	ATTTAAATATTTAAAATAT	446	10.07
Fatty acid metabolism					
*alr0240*	Malonyl coenzyme A-acyl carrier protein transacylase	C	AATTAAAAATTCAAAATGC	92	11.40
*asr3342*	Acyl carrier protein	C	AACAGTATTTACAAAATGT	309	10.49
*all1597* (*desB*)	Omega-3 fatty acid desaturase	C	TTCTGTAAATTCAATAAAT	299	10.25
Biosynthesis of siderophores					
***all2649***^c^	Non-ribosomal peptide synthetase	C	AATAGAAACTTGCAATAAT	110	10.23
Biosynthesis of amino acids					
*alr1244*	Shikimate kinase	C	TTAAAAATTTACATCAAGT	442	10.78
*all0414*	Anthranilate synthase	C	AAATATATCTTGCTTTAGT	314	10.66
Biosynthesis of cofactors					
***all1897* (*ho1*)**	Heme oxygenase 1	C	ATATAAATTCTCATCAAAT	107	14.34
*all5258*	Riboflavin synthase alpha chain	C	AAATAAAGTCAGAAAATAT	107	12.26
***alr1878* (*hemC*)**	Porphobilinogen deaminase	C	AATAGAAATCAGAAAATGT	122	11.08
Cell envelope					
*all2981* (*pbpH*)	Class A high-molecular-weight penicillin-binding protein	C	TAATATAATTGCAAAAAAT	81	10.93
*alr5066*	UDP-N-acetylenolpyruvylglucosamine reductase	C	TAAAAATTTCACAATTAAT	29	10.10
*alr0834* (*oprB-1*)	Porin; major outer membrane protein	C	AGAAAAATGCAGAAAATAT	57	10.01
DNA replication and repair					
*all1774* (*xseA*)^c^	Exodeoxyribonuclease VII large subunit	C	ATAAAAATATTCATAAAAC	375	12.36
*alr3812*	Mutator MutT protein	C	AGTTATATTTTTAATAAGT	252	11.84
*all7071*	Exodeoxyribonuclease V, alpha chain	alpha	AAAAAAAGTTACAATAATT	158	10.51
*all3632* (*avaIM*)	Site-specific DNA–methyltransferase	C	ATTATTATTTACAATAAAT	199	10.38
Transposon-related functions					
*all4465*	Transposase	C	TAATAAAGTTTGAATATAT	172	12.96
*all3682*	Transposase	C	CTTAAAAATTTGAATTTGT	354	10.55
*all8559*	Transposase	delta	AATTATAGTTGCAATTAAT	168	10.17
*alr8560*	RNA-directed DNA polymerase (reverse transcriptase)	delta	AATTATAGTTGCAATTAAT	642	10.17
*alr3384*	Transposase	C	AATAAAATTAACCAAAAAT	317	10.13

^a^Gene identification and protein description according to the cyanobacteria genome database CyanoBase (http://genome.microbedb.jp/cyanobase). Previously described FurA targets are indicated in bold letters.

^b^Annotated location of the ORF in the *Anabaena* sp. PCC 7120 genome: C, chromosome; alpha, beta and delta are referred to the natural plasmids pCC7120alpha, pCC7120beta and pCC7120delta, respectively.

^c^Genes contained in clusters, probable or already identified operons.

^d^Genes containing more than one predicted FurA box in their promoter regions.

As expected for the master regulator of iron homeostasis (25), predicted FurA-binding sites were identified in several genes related to iron uptake systems such as the previously recognized targets *alr0397* (*schT*), *alr3242* (*hutA2*) and *all1101*, all of them coding for TonB-dependent receptors, as well as the nine-gene cluster *all2649-all2641* encoding polyketide synthases and non-ribosomal peptide synthetases involved in siderophore biosynthesis (12). However, at least other 12 new putative FurA targets involved in transport across the cellular membranes were identified (Table 1), including an iron(III) dicitrate ABC transporter permease (*all2586*), a probable Zn^2+^/Fe^2+^ permease (*all0473*), the *znuAB* operon (*all0833-all0832*) encoding components of a high affinity zinc-uptake system (47), the ammonium transporter Amt4 (*alr0990*), and a cation-efflux system protein (*all2900*). Given the connection between iron homeostasis and oxidative stress, it was not surprising that candidate FurA-binding sites were associated to genes encoding proteins involved in defences against oxidative stress, such as the flavodiiron protein Flv3 (*all3895*) or the putative alkylhydroperoxidase encoded by gene *all5371.*

Notably, many of the genes associated with predicted FurA-binding sites encoded proteins involved in important regulatory functions, such as thioredoxin (*asl7641*), the bacteriorhodopsin Asr (*alr3165*) and the adenylate cyclases CyaD and CyaC (*all0743*, *all4963*). In addition, Fur-binding sites were found upstream of three genes encoding transcriptional regulators (*all1651*, *alr2595* and *all3903*), as well as in the promoter regions of at least other 10 genes encoding proteins involved in signal transduction mechanisms (Table 1).

As expected, photosynthesis and respiration contained several predicted FurA targets encoding iron-containing enzymes, e.g. NADH dehydrogenase (*all1127*, *alr0869*) or cytochrome *c* oxidase (*alr0950*). High-score FurA-binding sites were detected not only upstream of the iron-stress-induced flavodoxin (*isiB*), but also in front of the gene encoding the photosystem (PS) I subunit *psaK* (*asr4775*).

The *in silico* prediction also identified new putative targets of FurA involved in heterocyst differentiation, including the regulators *hetC* (*alr2817*), *patA* (*all0521*) and *patS* (*asl2301*). It is worth noting that the Fox gene *alr1728* (48) contains two predicted FurA-binding sites into its promoter region. Candidate FurA-binding sites were likewise predicted in different metabolic routes such as carbon fixation (*all4861*), the pentose phosphate cycle (*alr4670*), as well as the biosynthesis of fatty acids (*alr0240*, *all1597*), amino acids (*alr1244*, *all0414*), riboflavin (*all5258*) and peptidoglycan (*alr5066*). Curiously, putative FurA-binding sites were detected in several transposases (Table 1).

### Experimental validation of selected putative FurA-binding sites

To validate the functionality of the FurA-binding sites predicted by our probabilistic model, we selected 20-novel FurA targets candidates belonging to at least seven different functional categories (representing 20% of the total predicted targets with known function). In the experimental validation, we made special emphasis in putative novel targets involved in distinctive processes of cyanobacteria as microbial group, such as oxygenic photosynthesis, heterocyst differentiation or light-dependent signal-transduction mechanisms, among others. EMSA analyses were carried out using 300- to 400-bp-DNA fragments corresponding to the promoter regions of each selected gene, encompassing the predicted FurA-binding sites. To confirm the specificity of bindings, all assays included the promoter region of the *nifJ* gene as non-specific competitor DNA (31). The impact of metal co-repressor and reducing conditions on the *in vitro* affinity of recombinant FurA to its putative targets was evaluated in all assays. The specific binding of FurA to the promoter regions of its own gene (36) and *isiA* (49) were used as positive controls, while promoters of *Anabaena* sp. superoxide dismutases genes *sodA* and *sodB* were included as negative controls (33).

**Figure 1.**
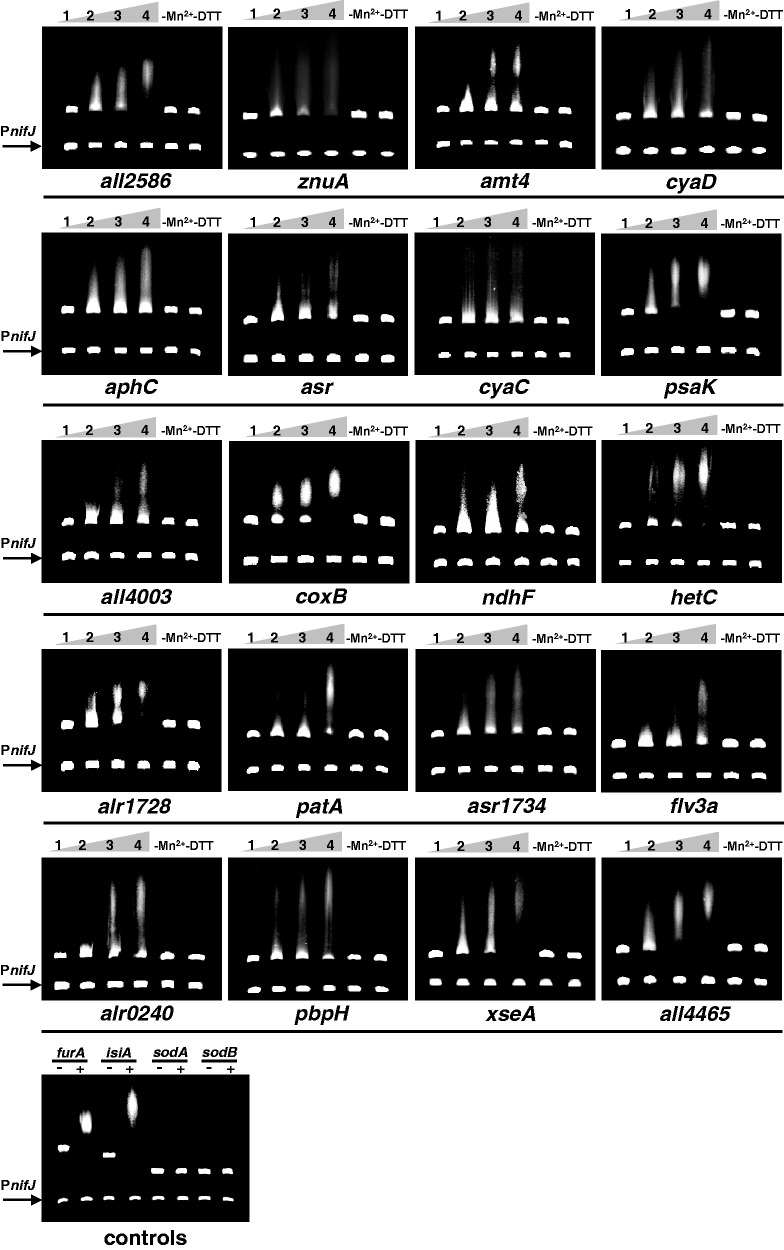
EMSAs showing the ability of FurA to bind *in vitro* the promoter regions of selected novel candidate target genes containing predicted FurA-binding sites. DNA fragments free (1) or mixed with recombinant FurA protein at concentration of 300 nM (2), 500 nM (3) and 700 nM (4) in the presence of Mn^2+^ and DTT were separated on a 4% PAGE. The impact of the metal co-regulator (removing Mn^2+^/adding EDTA) and reducing conditions (removing DTT) on the *in vitro* affinity of FurA (700 nM) to each target are also showed. The promoter region of *nifJ* gene was used as non-specific competitor DNA in all assays. Bindings of FurA (700 nM) to its own promoter, and to the *isiA* gene promoter were included as positive controls, while promoter regions of superoxide dismutases genes *sodA* and *sodB* were used as negative controls.

As shown in Figure 1, the EMSA experiments demonstrated that FurA specifically bound *in vitro* to all the promoter regions containing *in silico* predicted FurA-binding sites. All the tested DNA fragments were shifted in the presence of up to 700 nM FurA in a dose-dependent manner, whereas the same concentrations of the regulator were unable to shift either the non-specific competitor or both negative controls. As occurs with all so far described FurA targets, the *in vitro* specific binding of the regulator to the operator regions of these novel target genes was strongly dependent on the presence of divalent metal ions and reducing conditions. Thus, the EMSA results confirmed that DNA fragments containing the predicted Fur boxes for these 20 selected candidate targets were recognized *in vitro* by purified recombinant *Anabaena* sp. FurA and can therefore be considered as *bona fide* binding sites.

### FurA functions not only as a repressor, but also as an activator of gene expression

**Figure 2.**
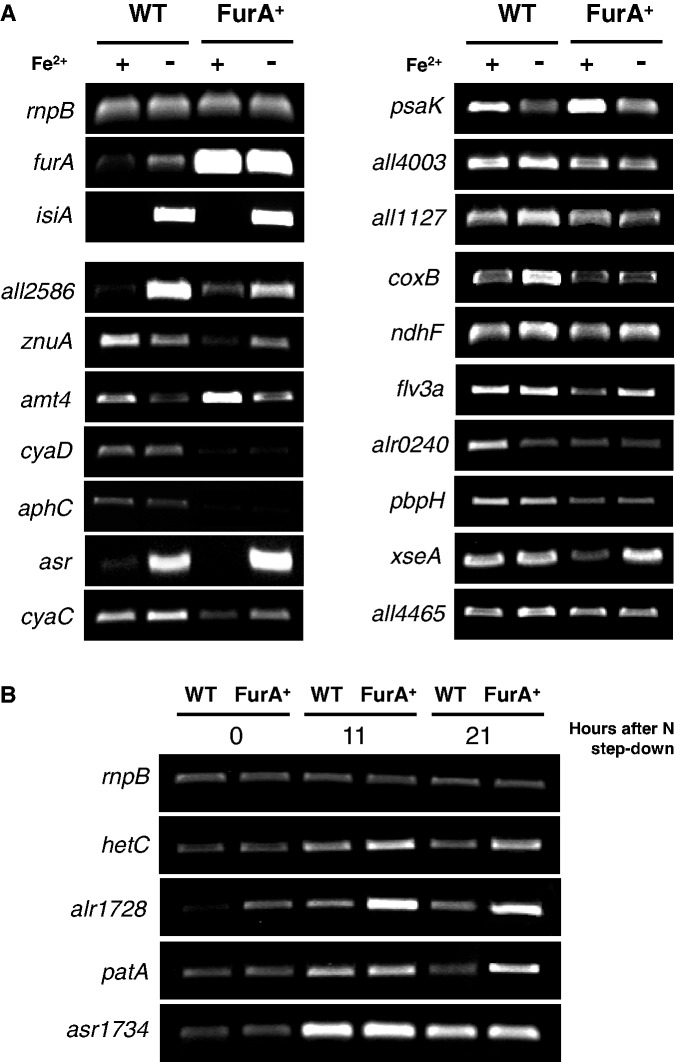
Semi-quantitative RT-PCR analyses showing the impact of FurA overexpression on the transcriptional pattern of several predicted FurA targets. (A) Total RNA from the wild-type strain PCC 7120 (WT) and the *furA* overexpressing strain AG2770FurA (FurA^+^) were isolated from cells grown in standard BG-11 medium (+Fe^2+^) or iron deprived medium BG-11_-Fe_ (–Fe^2+^). (B) In the case of candidate targets involved in heterocyst differentiation, RNA was isolated from ammonium-grown cells subjected to nitrogen deficiency under iron-replete conditions (BG-11_0_ medium) for the number of hours indicated. Housekeeping gene *rnpB* was used as control. Determinations for each gene were performed in the early exponential phase of PCR. Expression analyses of genes *furA* and *isiA* were included as controls of experimental conditions. All determinations were performed three times with independent biological samples, and the relevant portion of a representative gel is shown for each gene. Relative induction ratios are shown in Supplementary Tables S2 and S3.

In order to analyse the effect of FurA on gene expression *in vivo*, the set of 20 selected novel FurA targets previously validated by EMSA were divided in two groups. A major group included genes with an expected transcriptional response to iron deprivation, comprising those encoding proteins involved in transport across the membrane, PS subunits, respiratory iron-containing enzymes, signal transduction mechanisms and others. The impact of iron availability and FurA overexpression on the transcriptional pattern of these selected genes was determined by sqRT-PCR (Figure 2A). As control of iron deprivation, we included in the transcriptional analysis the iron-stress induced gene *isiA*, which have been identified as FurA target in previous studies (49). A second group of genes included novel predicted and EMSA-validated FurA targets involved in heterocyst differentiation, whose expression is developmentally regulated under nitrogen deficiency and therefore, their transcript abundances in vegetative cells growing in media containing combined nitrogen is only basal (4). Since FurA appears naturally induced in proheterocyst during the middle to late stages of differentiation (30), we analysed by sqRT-PCR the influence of FurA overexpression on the transcriptional patterns of four predicted novel targets after 11 and 21 hours of nitrogen step-down (Figure 2B).

As shown in Figure 2A, the expression of most FurA targets analyzed in the first group appeared induced under iron deprivation in the wild-type strain, even the iron-containing enzymes cytochrome *c* oxidase (*coxB*) and NADH dehydrogenases (*all1127*, *ndhF*), a fact that presumably occurred as a consequence of the metal co-repressor scarcity. However, despite the *in vitro* affinity of FurA strongly depended in all cases of the presence of metal co-repressor (as previously shown in EMSA analysis), the expression of several target genes like *znuA*, *amt4*, *aphC*, *psaK*, *alr0240* and *pbpH* did not increase but rather decreased under iron deprivation (Figure 2A and Supplementary Table S3). Decrease in transcript abundance of such FurA direct targets under iron limitation could reflect an iron-dependent transcriptional activation by FurA, a fact previously observed with other FurA targets (25), or be the result of an opposite co-regulation by other transcription factors.

Overexpression of FurA in *Anabaena* sp. led to discern two distinguishable patterns of transcriptional regulation. Most of the selected target genes appeared downregulated to a greater or lesser extent in a FurA overexpression background under iron-replete conditions. However, at least two genes (*amt4* and *psaK*) were clearly induced under the same environment (Figure 2A and Supplementary Table S3), suggesting an iron-dependent transcriptional activation of FurA on the expression of these novel targets. A slightly inductive effect of FurA overexpression was also observed in the ferric-dicitrate permease encoding gene *all2586* (Figure 2A, Supplementary Table S3), although this fact was relatively expected according to our experience, since a similar behaviour has been observed with all the iron metabolism players so far described as FurA targets (25,33). It has been hypothesized that Fur proteins might act as ferrous ion buffers into the cell by increasing the Fe^2+^-binding capacity of the cytosol during oxidative stress (17). We speculate that the high level of FurA expression achieved by the *Anabaena* sp. strain AG2770FurA (33) could suddenly reduce the intracellular free iron pool, leading to the release of metal co-repressor from some FurA–Fe^2+^ complexes and therefore allowing the transcription of most sensible iron-responsive target promoters.

In most cases, the depletion of metal co-regulator mitigates the transcriptional effect of FurA overexpression, either when the protein acted as repressor or as activator of gene expression. Some FurA-repressed targets including *asr*, *cyaC*, *flv3a* and *xseA* displayed higher induction levels due to iron limitation in the *furA*-overexpressing strain that those observed in the wild-type strain as the result of the same nutritional deficiency. In fact, for few of these targets such as *asr* and *xseA*, the overexpression of FurA seemed to exert a synergistic inductive effect to iron starvation on gene transcription (Figure 2A and Supplementary Table S3). It was remarkable the strong induction of *Anabaena* bacteriorhodopsin Asr under iron starvation, even in a FurA overexpression phenotype.

The influence of FurA overexpression on the pattern expression of several targets involved in heterocyst differentiation was also evaluated. Since iron deprivation severely impairs heterocyst differentiation (50), the transcriptional response of this second group of genes was only analyzed under iron-replete conditions. As shown in Figure 2B and Supplementary Table S4, the overexpression of the metalloregulator led to a clear increase in transcript abundance of *hetC*, *alr1728* and *patA*, while influence on *asr1734* transcription level was quite weak. However, the slight induction of the heterocyst differentiation regulator Asr1734 could be in fact the result of a FurA-mediated transcriptional activation, maybe diminished or modulated by the variety of other co-acting signals that influence the heterocyst development (4).

## DISCUSSION

Computational approaches have proven quite useful for identifying *cis*-acting regulatory elements that function as binding sites for transcription factors (51,52). These strategies have been successfully used to expand the knowledge of multiple regulons, from microorganisms to humans (53,54), including those associated to several Fur proteins (55–58). In this article, we scan the *Anabaena* sp. PCC 7120 genome in the search for FurA putative binding sites matching the position weight-matrix generated from a data set comprised of foot-printed sites. Predicted FurA-binding sites were identified upstream of 215 genes belonging to diverse functional categories, which represent 3.4% of the open reading frames (ORFs) annotated in the *Anabaena* sp. PCC 7120 genome (41). Even without taking into account that possible false positives can be included in our prediction, the magnitude of the FurA-predicted regulon resembles those of other Fur-regulatory networks previously described in some non-photosynthetic bacteria. Almost 10% of genes in the *Neisseria gonorrhoeae* genome responded to iron availability with 30% of those ORFs regulated directly by Fur (59), while Fur directly or indirectly regulated ∼6.5% of the *Salmonella typhimurium* genome (60). Distinctive and highly iron-consuming cyanobacterial processes such as oxygen-evolving photosynthesis or nitrogen fixation, among others, undoubtedly expands the scope of Fur-regulated genes in cyanobacteria, as compared with most heterotrophic prokaryotes.

**Figure 3.**
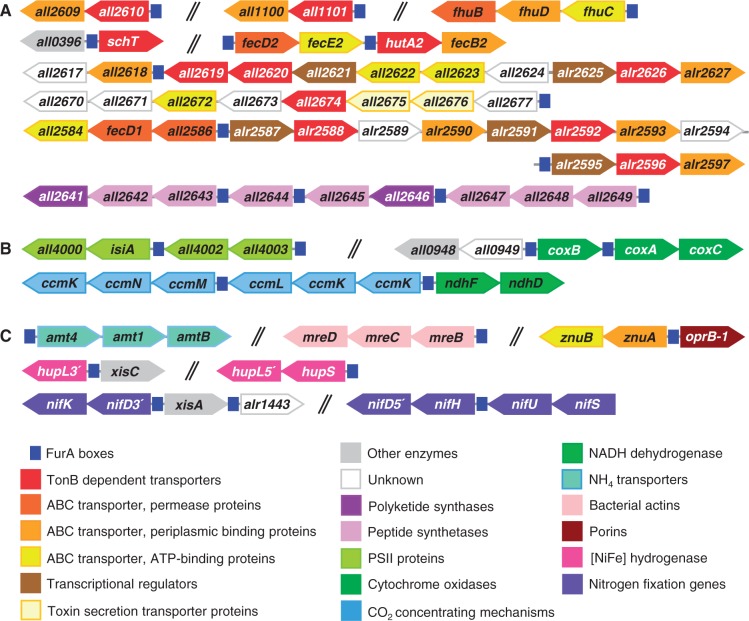
Some predicted and/or experimental validated FurA-regulated gene clusters and location of associated FurA boxes. Gene clusters have been grouped in three main categories: (A) iron metabolism, (B) photosynthesis and respiration and (C) other cellular processes. Boxes representing each gene have not been drawn to scale and indicate direction of transcription. The double-hashed lines separate clusters which are not contiguous in the genome. The nomenclature of the color code is indicated.

Our weight-matrix-based prediction model proved to discern FurA boxes from non-cognate sequences, while EMSA experiments confirmed *in vitro* specific binding of the regulator to selected 20% of predicted targets with known function. It is interesting to note that the affinity of Fur proteins for DNA is not the same in all their regulatory sequences (57). Our cutoff value was selected to reduce biases introduced by false positives, and might intrinsically overlook weak binding sites that significantly diverge to those experimentally identified which may not accurately reflect the statistical distribution of *bona fide* sites. On the other hand, many of the FurA-binding sites predicted here, and even some of the FurA boxes experimentally recognized in previous studies are located in promoter regions of putative clusters, operons or divergent genes (Figure 3). Therefore, it is reasonable to speculate that our determinations underestimate the actual magnitude of the FurA regulon.

The results presented here and previous results (25) suggest that *Anabaena* sp. PCC 7120 FurA presumably associates with DNA at a 19- to 23-bp consensus binding site with the sequence 5′-AAATAAATTCTCAATAAAT-3′, which shares 58% homology to the *E.coli* Fur box consensus sequence (61) and 52–68% homology to the Fur-binding consensus sequences from other eubacteria (47,62–69) (Supplementary Figure S2A). Considering the different models proposed to discern the stoichiometry of Fur–DNA interactions (18,61), FurA could recognize its consensus as 8-mer inverted motifs (25) or following a 7-1-7 model (Supplementary Figure S2B), as proposed by Napolitano and co-workers with the FurB/Zur paralog from *Anabaena* sp. (47). Overlapping or contiguous AT-rich motifs located upstream or downstream of the FurA-box sequence would allow the association of additional protein dimers to less-conserved DNA-binding sequences, as observed in previous DNase I protection assays (25,34,36). Minor sequence changes among natural FurA boxes in combination with different ranges of cooperative binding and the possible simultaneous action of other transcriptional regulators could explain the differences observed in the expression of the FurA-regulated genes, either repressed or induced, in response to the same environmental stimulus (18). The large number of genes regulated by Fur as well as its tendency to polymerize along the DNA are supported by an atypical abundance of this regulator into the cell (17), which might also be related to moonlighting functions (70).

We have previously documented that FurA plays a central role in iron homeostasis in *Anabaena* sp. PCC 7120 (25). FurA boxes have been experimentally confirmed in at least nine clusters of genes involved in siderophore biosynthesis and ferri-siderophore transporting systems (Figure 3A), as well as in genes related to iron storage mechanisms. Most of these targets primarily respond to iron availability and appeared transcriptionally repressed by FurA under iron-sufficient conditions (25,33). Notably, several of these FurA-regulated clusters contain more than one predicted and/or experimental validated binding site located at different intergenic regions throughout the cluster. A typical example is the cluster of nine ORFs (*all2641*–*all2649*) encoding seven non-ribosomal peptide synthetases and two polyketide synthases involved in the biosynthesis of siderophores (12). Previous analyses have shown that this nine-gene cluster contains four FurA boxes, a FurA box located upstream to the entire cluster and three other FurA boxes within downstream promoters. EMSA analyses showed a higher affinity of recombinant FurA to the upstream FurA box, though *in vitro* binding to all the three downstream promoters also occurred but with less affinity (25). This multi-targeted arrangement with different DNA-binding affinities allows a sequential modulation of the cluster and a graded expression of the gene products in relation to the magnitude of the environmental signal that controls the activity of the transcriptional regulator, in this case the iron status (71). In other cases, FurA boxes appear located within bidirectional promoters (Figure 3). These locations might allow the simultaneous control of opposing genes/clusters by interacting with two overlapping operators (72,73).

Our findings further support not only the role of FurA as a dual regulator in cyanobacteria, acting both as a transcriptional repressor but also as an activator of gene expression. FurA appears to induce in a greater or lesser extent the expression of at least six novel targets (*psaK*, *amt4*, *hetC*, *alr1728*, *patA* and *asr1734*). As we could infer from the *in vitro* DNA-binding assays, the FurA transcriptional modulation depended in all cases on the presence of metal co-regulator and reducing conditions. Despite Fur-mediated direct activation as been observed in several heterotrophic bacteria (20,21,24,74), little is known about the mechanism or mechanisms by which Fur proteins function to directly activate gene transcription. In *N.gonorrhoeae* (20), Fur-mediated activation occurs by competition to another repressor for overlapping binding sites, resulting in derepression of transcription. Interestingly, the locations of the Fur boxes in the promoters of these Fur-activated genes were close to the −10 and −35 motifs, which could suggest not only the competition with other repressors, but also the possibility of RNA polymerase recruitment to enhance transcription initiation. When Fur boxes are located far upstream of the transcription start site, Fur might activate gene expression by altering DNA morphology allowing or enhancing RNA polymerase binding (21).

The *in vitro* affinity of recombinant FurA to the target sequences experimentally validated so far strongly depended on the presence of divalent metal ions and reducing conditions, suggesting a critical role of metal co-repressor and the redox status of the cysteines to the function of this metalloregulator *in vivo* (35,36). However, several genes analyzed here appeared overrepressed under a FurA overexpression background and iron-replete conditions but were not induced in the wild-type strain as response to iron deprivation. It is likely that gene expression of those FurA targets (*znuA*, *cyaD*, *aphC*, *alr0240*, *pbpH*) are co-modulated by other transcriptional regulators that have the opposite effect to FurA under iron limitation. Hence, downregulation of possible activators or the induction of another repressor under low-iron environment could explain the transcriptional response observed in these FurA-regulated genes. That is the case of *znuAB* operon, which encodes components of a high-affinity zinc uptake mechanism and is co-regulated in *Anabaena* sp. PCC 7120 by FurB/Zur (47). We speculate that co-action of both regulators on zinc uptake mechanisms could be related to protection of cells under oxidative stress. Oxidative stress could led to release of Zn^2+^ from thiols triggering the elevation of intracellular free zinc pool, and under this condition the additional uptake of zinc could exacerbate the risk of oxidative damage (75). Oxidative stress induces not only the expression of FurB in *Anabaena* sp., but also FurA expression (29) in order to repress iron uptake and limit Fenton reactions (76). The presence of FurA boxes on *cis*-acting regulatory sequences of zinc uptake mechanism might allow an additional switch-off under oxidative stress.

As many other filamentous diazotrophic cyanobacteria, *Anabaena* sp. PCC 7120 develops a one-dimensional pattern of specialized nitrogen-fixing cells called heterocysts when grows in combined nitrogen-deprived environments, in order to spatially separate the highly oxygen-sensitive nitrogenase from the oxygen-evolving photosynthesis. Heterocyst development and its pattern formation are developmentally regulated processes, involving the coordinated action of several transcriptional regulators which orchestrated a complex regulatory cascade (4,5). Our previous analyses have shown that expression of *furA* is strongly induced by the global regulator of nitrogen metabolism NtcA in proheterocysts during the first 15 h after nitrogen step-down, remaining stably expressed in mature heterocysts (30). On the other hand, *in vitro* and *in vivo* analyses have shown that FurA acts as a transcriptional repressor of the *ntcA* expression (34). Taken together, the data appeared to suggest that FurA might function as an NtcA shutoff switch, which in conjunction with other signals regulates the timing of NtcA induction during the heterocyst development. The results presented here clearly indicate a direct activating role of FurA on the expression of other players involved in heterocyst differentiation, such as those encoding by genes *hetC*, *patA* and *alr1728*, while also suggest the modulation of other predicted targets like *asr1734* or *patS.* These data not only support the connection between iron homeostasis and heterocyst differentiation via FurA, but also demonstrate that this global regulator may exert a dual action even on the same physiological process or metabolic route, a fact previously observed in the tetrapyrrole biosynthesis pathway (25).

Our *in silico* analysis and experimental determinations not only underlined the role of FurA in cyanobacterial distinctive processes like photosynthesis and heterocyst differentiation, but also revealed Fur regulatory functions on physiological processes not previously described in heterotrophic bacteria, such as light-dependent signal-transduction mechanisms. Sensing light signals and their subsequent transduction is essential for photosynthetic organisms, since it enables them to adapt to variable environmental conditions (77). FurA-binding sites were predicted and functionally validated in the promoter regions of both *aphC* and *cyaC*, indicating at least an indirect control of Fur on the expression of genes modulated by this cAMP signal transduction cascade (78). Likewise, FurA-binding sites were detected upstream of genes encoding the *Anabaena* sensory rhodopsin Asr (79), as well as the adenylate cyclase CyaD (80). Curiously, Asr appeared strongly induced under iron-limited conditions, a fact that could suggest a possible role of Asr as a low-iron demanding light-driven photon pump when *Anabaena* faces iron-poor environments (81,82). Overall, these data greatly enhance the complexity and extend the range of the FurA regulatory network in *Anabaena* sp., highlighting novel functions of Fur proteins in cyanobacteria.

Notably, insertion sequences (ISs) constitutes ∼2.4% of the protein-encoding genes in *Anabaena* sp. PCC 7120 (83). ORFs associated to these transposable elements encode transposases, enzymes that mediates the movement of the concerned sequence within the genome (84). Transposition of ISs must be tightly regulated, since the DNA rearrangements that they cause, including gene inactivation or deletions, could be disadvantageous or even lethal under ‘normal’ conditions. However, transposition may provide evolutionary advantages in certain situations, when activation or formation of new genes enhances cell survival allowing the adaptation to new environmental situations, including nutritional stresses (85). Transcription of the transposase-encoding gene *all4465* increased under iron deprivation and appeared repressed by FurA in a metal and reducing conditions dependent manner. The occurrence of predicted Fur boxes upstream of a number of transposase genes could suggest a regulatory role of FurA on transposition linked to iron starvation or environmentally induced oxidative stress in *Anabaena* sp. Further studies will be required to verify this hypothesis.

In summary, the results presented here significantly expand our understanding of the FurA regulon in *Anabaena* sp. PCC 7120, a filamentous cyanobacterium commonly used as a model organism for studying processes such as photosynthesis, nitrogen fixation, cell differentiation and multicellularity in prokaryotes. Our genome-wide prediction of candidate FurA-binding sites supported by experimental validations is particularly relevant given the potentially essential role of this metalloregulator in the physiology of *Anabaena* sp. (35), which impairs classical genetic approaches to study Fur regulatory functions. Our analyses unravel the role of FurA as a global transcriptional regulator, acting both as repressor and activator of gene expression. In either case, the *in vivo* FurA-mediated regulation seems to be dependent of the environmental iron availability as well as the intracellular redox status. Thus, FurA appears to couple iron homeostasis and oxidative stress to major physiological processes in cyanobacteria.

## SUPPLEMENTARY DATA

Supplementary Data are available at NAR Online.

## FUNDING

Ministerio de Ciencia e Innovación, Spain [BFU2009-07424 and BFU2010-16297]; Ministerio de Economía y Competitividad, Spain [BFU2012-31458]; Consejo Superior de Investigaciones Científicas (doctoral fellowship to V.E.A.). Funding for Open Access: MINECO [BFU2012-31458].

*Conflict of interest statement*. None declared.

## Supplementary Material

Supplementary Data
