# Supplementary material for: The FurA regulon in Anabaena sp. PCC 7120: in silico prediction and experimental validation of novel target genes
Source: Nucleic Acids Res. 2014 Feb 6;42(8):4833–46. doi: 10.1093/nar/gku123 (PMC4005646; doi:10.1093/nar/gku123)
Supplement: Supplementary Data [file supp_42_8_4833__index.html]

The FurA regulon in Anabaena sp. PCC 7120: in silico prediction and experimental validation of novel target genes — The FurA regulon in Anabaena sp. PCC 7120: in silico prediction and experimental validation of novel target genes — Supplementary Data 

# The FurA regulon in *Anabaena* sp. PCC 7120: *in silico* prediction and experimental validation of novel target genes

## Supplementary Data

files

**Files in this Data Supplement:**

- Supplementary Data - pdf file
- Supplementary Data - xls file
