# Supplementary material for: The FurA regulon in Anabaena sp. PCC 7120: in silico prediction and experimental validation of novel target genes
Source: Nucleic Acids Res. 2014 Feb 6;42(8):4833–46. doi: 10.1093/nar/gku123 (PMC4005646; doi:10.1093/nar/gku123)
Supplement: Supplementary Data [file supp_gku123_nar-02945-v-2013-File007.pdf]

Table S1. Oligonucleotides used in this study

Primer	Sequence 5´- 3´
EMSA	
Pall2586_up	GCCGTGCCATCTTTGGCATAG
Pall2586_dw	CATTATCTCCTAGTTAATTG
PznuA_up	GGGTTAACCAGCCAATACTTCC
PznuA_dw	CTGATGTGAGTCCAAATCACGG
Palr0990_up	CTATCCGATGGGAGTTGATTATG
Palr0990_dw	GATGTTAGTAGTAGCAGACTTACCC
PcyaD_up	CTCAGTATCTAAATTTAGATAATGCTCATG
PcyaD_dw	GGGTGTAATATATGTATCCTGGCAGATG
PaphC_up	GAAGACACGATGTTAAGCGTG
PaphC_dw	GATTGAGACTAACCTGAGAATTTGG
Pasr_up	GCTCCGTGATGGGATAAGCAC
Pasr_dw	GACTCTCCAAATTCATAGGGC
PcyaC_up	CCCTAATTGCTGTCCTCTTACTC
PcyaC_dw	GGTTGTCTGGCTCATCATCAAC
asr4775-1	CGCTCAACCACTCGTCCAC
asr4775-2	CAACTGTAGGACTCCATTCC
all4003-1	CGTGCAGTAATGACAGAGAG
all4003-2	CACAGCGATGCTCGTCATC
alr0950-1	CATTACCGACCCATCTCCAC
alr0950-2	GTAATGTCCAGATTGAACTTGG
P2-ndhF_up	GCGAGGAGTGTTCAGAGGC
P2-ndhF_dw	GGCACACACCAACTTGTTGC
PhetC distal_up	GCTACTAGAAATGAGGAGAGGG
PhetC distal_dw	GTGAGCAACATCGACATCTG
Palr1728_up	CAGTTTTAATGAATACCCAATAAG
Palr1728_dw	CCTCATTGTGACAAGTATTCTCAC
PpatA_up	GGTTTGAGCCAATAACTATACGTG
PpatA_dw	GTAATCGGAAGTGTTTTCATGG
P2asr1734_up	GATTTTACTTTTGATAAAATTGCTC
P2asr1734_dw	CGAAGATTGAATCTTCCTGTGTC
Pflv3a_up	CCTTTTCCTCGCTTCCCACTTC
Pflv3a_dw	CTCGGTGAGTGCTACCATAACTCTC

Palr0240_up	CTTGATACTGTAGCTCAACGCC
Palr0240_dw	CCAGTGCTTGAGAACCTTGTCC
PpbpH_up	CGCATTGGTATGCTATGACC
PpbpH_dw	GGCGATTTCACTTCCTCACTTG
PxseA_up	GAATCGCCAGAAGCAGACAAATG
PxseA-dw	CAAGTCTATCCAAATCGTAGCATTG
Pall4465_up	CGTAATGGAGTCGGCAATTTG
Pall4465_dw	CGTGGACAAATCCTACGC
PfurA_up	CTCGCCTAGCAATTTAACAAC
PfurA_dw	GCCTTGAGCGAAGTATTTGTC
PisiA_up	CAGATTGTCTGTTGAGACTG
PisiA_dw	GCTACGTGTGCGCCGATG
PsodA_up	GATAGAATCAATGACTGCTC
PsodA_dw	GTTGTTGCCTCTTATTTG
PsodB_up	GCTTAGTAGTCCCTTTGC
PsodB_dw	CATTTTGTGAAGTCCTCTC
PnifJ_up	GCCTACTCTGCGAGTTCTCCG
PnifJ_dw	GGCCTGTGAGAGTTGCTGCAC
RT-PCR	
all2586-RT_up	GAATCCTTTATCACCTTTGATG
all2586-RT_dw	CACCCAAGCAGTCTGTTGAC
znuA-RT_up	GTTGTCCATGACGGTTGGTTG
znuA-RT_dw	GCCTCAGCATTAGCTTGATAGG
amt4-RT_up	GCGGTAGATACTCCTACCCTAGA
amt4-RT_dw	GCCTAACCAACCATTGGAG
cyaD-RT_up	CGAGATGTGTTCACAATTGGTCG
cyaD-RT_dw	GCTGAGTTCTTCTTCACCATCTC
aphC-RT_up	CCGTTTGGAACGACACCTAC
aphC-RT_dw	CACTAGGGTTGGGTAATTCTTGC
asr-RT_up	CCAGTACGAATACCTTGTGGCG
asr-RT_dw	GGGTAGCCTATCCAAAGCACTG
cyaC-RT_up	GCACGCTGCGTCAAGAGTC
cyaC-RT_dw	GCTGTTGTAATGGTATTGATCAAGG
psaK-RT_up	CCTTACTCGCTGCTGCAAC
psaK-RT_dw	GATTCTGCCTAGATTATGCAATCC

all4003-RT_up	GCCCGTTTCCAGACATCAC
all4003-RT_dw	CCACCAACCAAGTCTTCGAG
all1127-RT_up	CCCTACGACACCTTAATTGTCG
all1127-RT_dw	GCCCATAATACAGTCTTGGAGG
coxB-RT_up	CAATGATGACCGTTTCCGC
coxB-RT_dw	CACCAGTGGTGATACCTGACTCAG
ndhF-RT_up	GCGTTGGAACTCTCACCTG
ndhF-RT_dw	GCAACCACCAGTGAGTTCCG
flv3a-RT_up	GCTAATCAATCCCACAGAGATC
flv3a-RT_dw	GCTAGGCGATCGCTATACCC
alr0240-RT_up	CGGACAAGGTTCTCAAGCACTG
alr0240-RT_dw	CTCTACAGCAGCCGTATTCATG
pbpH-RT_up	CGCAACGTCGTACTTCGTC
pbpH-RT_dw	GACCCCAATACACTTGATTGAG
xseA-RT_up	CACTTCAAGATCCTGATGGTTCCG
xseA-RT_dw	CGTCGGTGTATGCACACAAACATC
all4465-RT_up	CCAAGCTTCTATCGAACGGG
all4465-RT_dw	CCAGCATCATTAATAGATTTAGCG
FurA_up	CGGGATCCATGACTGTCTACACAAATAC
FurA-5last_dw	ACGCGTCGACCTAACGTTGGCACTTGGG
IsiA_up	CTGCTCTGACAACCCTCTGG
IsiA_dw	CAGCTAACTTGACTGTATCGGC
rnpB_up	AGGGAGAGAGTAGGCGTTG
rnpB_dw	AAAAGAGGAGAGAGTTGGTGG
hetC-RT_up	GGATTTGGCAAGAGCGATCG
hetC-RT_dw	CCACCTCAACGGGCGCTTC
alr1728-RT_up	GCTGTGGTTCGTCCCGTTGCTTC
alr1728-RT_dw	GCTTTGAGTAATTCTTCCATTAAGC
patA-RT_up	CTGGGAAGACAATCACTGGTTGTC
patA-RT_dw	GATATCTGGCTTTGTACAGAGAATTTCC
asr1734-RT_up	GAACTCGAAAGCATTACCACGG
asr1734-RT_dw	GAAGGCAATTATGTTAGTGAATTTC

Table S3. Relative induction ratios of selected FurA predicted targets under different experimental conditions, as result of semi-quantitative RT-PCR analyses[a]

ORF ID[b]	Gene symbol[b]	Protein description[b]	$WT_{(-Fe)}$ / $WT_{(+Fe)}$	$FurA^{+}_{(+Fe)}$ / $WT_{(+Fe)}$	$FurA^{+}_{(-Fe)}$ / $WT_{(+Fe)}$	$FurA^{+}_{(-Fe)}$ / $FurA^{+}_{(+Fe)}$
all1691	*furA*	Ferric uptake regulator,	4.52 ± 0.15	30.21 ± 1.15	29.17 ± 1.12	0.94 ± 0.09
all4001	*isiA*	Flavodoxin, iron-stress induced protein	35.12 ± 1.35	1.01 ± 0.03	36.23 ± 1.17	35.62 ± 1.12
all2586		Iron (III) dicitrate ABC transporter permease	38.58 ± 1.09	1.25 ± 0.27	20.14 ± 0.93	15.23 ± 0.86
all0833	*znuA*	Periplasmic binding protein ABC transporter	0.45 ± 0.06	0.17 ± 0.02	0.49 ± 0.08	3.12 ± 0.14
alr0990	*amt4*	Ammonium transporter	0.32 ± 0.08	2.81 ± 0.23	0.72 ± 0.11	0.29 ± 0.07
all0743	*cyaD*	Adenylate cyclase	0.95 ± 0.12	0.09 ± 0.02	0.15 ± 0.04	1.42 ± 0.09
all2699	*aphC*	Two-component sensor histidine kinase	0.67 ± 0.09	0.07 ± 0.01	0.12 ± 0.01	1.56 ± 0.06
alr3165	*asr*	Bacteriorhodopsin	23.12 ± 1.02	0.02 ± 0.01	33.8 ± 1.06	52.45 ± 1.12
all4963	*cyaC*	Adenylate cyclase	1.30 ± 0.15	0.23 ± 0.04	0.64 ± 0.09	2.57 ± 0.16
asr4775	*psaK*	Photosystem I subunit PsaK	0.36 ± 0.09	2.13 ± 0.06	0.62 ± 0.07	0.41 ± 0.05
all4003		Photosystem II CP43 protein PsbC homolog	1.14 ± 0.16	0.72 ± 0.04	0.43 ± 0.07	0.68 ± 0.06
all1127		NADH dehydrogenase	1.43 ± 0.12	0.89 ± 0.09	0.66 ± 0.08	0.72 ± 0.05
alr0950	*coxB*	Cytochrome *c* oxidase subunit II	2.35 ± 0.21	0.51 ± 0.08	0.59 ± 0.06	1.23 ± 0.08
alr0869	*ndhF*	NADH dehydrogenase subunit 5	1.47 ± 0.15	0.72 ± 0.03	1.18 ± 0.07	1.67 ± 0.06
all3895	*flv3a*	Flavodiiron protein Flv3	1.21 ± 0.08	0.34 ± 0.05	0.87 ± 0.11	2.39 ± 0.08
alr0240		Malonyl coenzyme A-acyl transacylase	0.36 ± 0.06	0.45 ± 0.06	0.29 ± 0.04	0.78 ± 0.05
all2981	*pbpH*	Penicillin-binding protein	0.82 ± 0.07	0.31 ± 0.06	0.43 ± 0.08	1.42 ± 0.06
all1774	*xseA*	Exodeoxyribonuclease VII large subunit	1.11 ± 0.05	0.47 ± 0.08	1.26 ± 0.04	2.83 ± 0.11
all4465		Transposase	1.51 ± 0.09	0.93 ± 0.03	0.89 ± 0.06	0.92 ± 0.04

[a]Signal assigned to each gene corresponded to the intensity of DNA band in the agarose gel stained with ethidium bromide, normalized to the signal observed for housekeeping gene *rnpB* in each condition. Values are means of three independent determination ± SD.
[b]Open reading frame identification, gene symbol and protein description according to the cyanobacterial genomes database CyanoBase (http://genome.kazusa.or.jp/cyanobase).

Table S4. Relative induction ratios of several FurA predicted targets involved in heterocyst differentiation at different times after nitrogen step-down, as result of semi-quantitative RT-PCR analyses[a]

**ORF ID**[b]	**Gene symbol**[b]	**Protein description**[b]	**FurA$^+$ / WT**[c]		
			**0 h**	**11 h**	**21 h**
alr2817	*hetC*	Heterocyst differentiation protein	1.37 ± 0.11	1.64 ± 0.07	2.46 ± 0.12
alr1728		Fox gene with unknown function	2.72 ± 0.08	3.96 ± 0.10	3.12 ± 0.17
all0521	*patA*	Heterocyst pattern formation regulator	1.27 ± 0.04	1.59 ± 0.04	3.28 ± 0.09
asr1734		Heterocyst differentiation negative regulator	1.21 ± 0.06	1.17 ± 0.03	1.13 ± 0.02

[a]Signal assigned to each gene corresponded to the intensity of DNA band in the agarose gel stained with ethidium bromide, normalized to the signal observed for housekeeping gene *rnpB* in each condition. Values are means of three independent determination ± SD.
[b]Open reading frame identification, gene symbol and protein description according to the cyanobacterial genomes database CyanoBase (http://genome.kazusa.or.jp/cyanobase).
[c]Both strains were grown by triplicate in BG-11 medium to mid-log phase, washed with BG-11_0 and resuspended in the same nitrogen free medium to induce heterocyst differentiation.

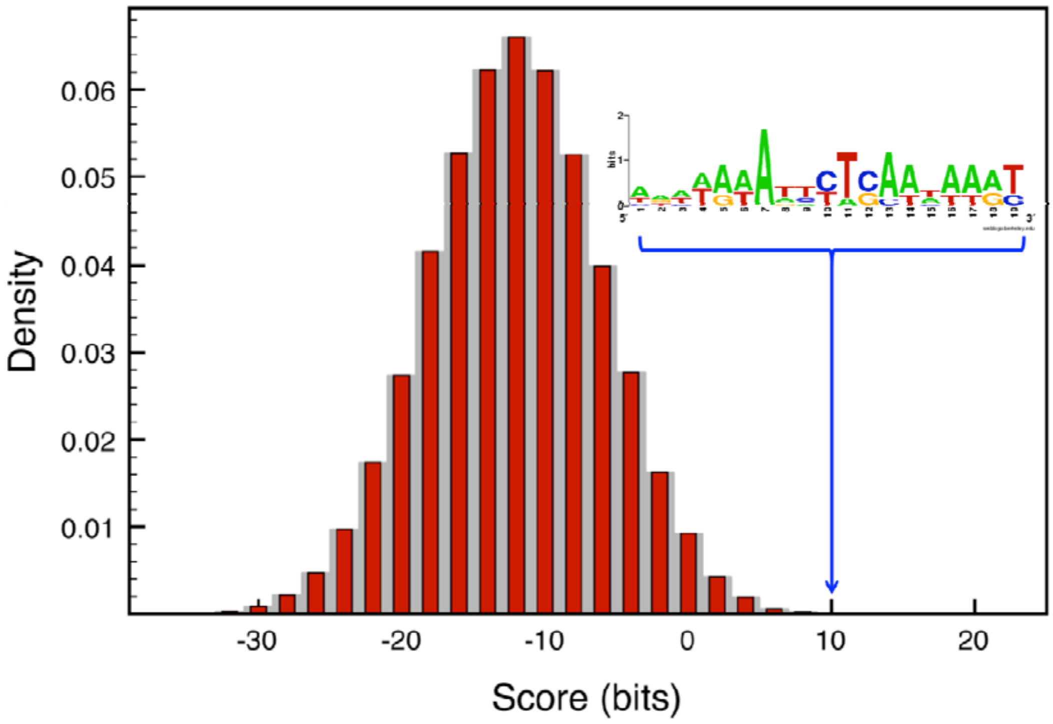

Figure S1. Benchmarking test in order to verify the reliability of the FurA weight-matrix to discern cognate and non-cognate binding sites. The matrix generated was used to scan all the non-coding sequences lying between two consecutive convergent genes found in the *Anabaena* sp. PCC 7120 genome. The scores resulting from this analysis are represented as a density histogram with a mean value around -11 bits. With a score range between -41.7 and 17.2 bits, the matrix scored unrelated sequences displaced towards negative values, while binding sites experimentally recognized by FurA scored at higher values, close to the matrix maximum score. The logo of the matrix used to scan the sequences was created with the WebLogo server (http://weblogo.berkeley.edu/), and it is included in the figure. The score value used as cutoff in the subsequent scanning of the *Anabaena* sp. PCC 7120 genome (10 bits) is highlighted. For this cutoff value, the $\wp$-value calculated parametrically for the score distribution histogram of sequences between convergent genes was 7.43 10-5 for a significance value of 0.01%.

A

Bacterial species	Fur box consensus	bp	Homology to FurA box
***Anabaena* sp. PCC 7120 FurA**	**-------AAATAAATTCTCAATAAAT-----**	**19**	
Anabaena sp. PCC 7120 FurB	------AATAATGATAATTATTATCAATAAA	25	(68.42)
Mycobacterium tuberculosis	CATATTGAAAATCATTTTCATCAAAC-----	26	(68.42)
Vibrio cholerae	------GATAATGATAATAATTATC------	19	(63.16)
Bacillus subtilis Fur	-----------TGATAATNATTATCA-----	15	(60.00)
Escherichia coli	------GATAATGATAATCATTATC------	19	(57.89)
Pseudomonas aeruginosa	------GATAATGATAATCATTATC------	19	(57.89)
Neisseria gonorrhoeae	------AAAATTTATAATTTTTAAC------	19	(57.89)
Bacillus subtilis PerR	-----------TTATAATNATTATAA-----	15	(53.33)
Campylobacter jejuni	------TATTTGATAATTATTATCA-----	20	(52.63)
Neisseria meningitidis	-------GATAATATAATAATTATCTTT---	21	(52.63)

B

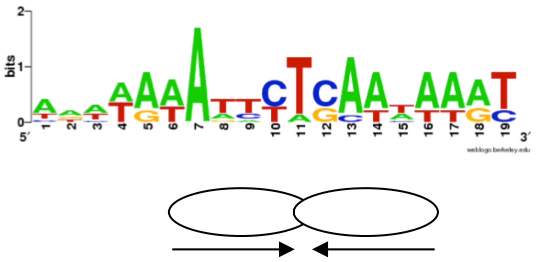

AAATAAATTCTCAATAAAT consensus
ATAAATATTCTCAATAAGC *furA1*
TGTTATATTCTCAATTAAC *furA2*
ATATAAATTCTCATCAAAT *ho1*
AATAGAAATCAGAAAATGT *hemC*
CAAAATAGTCTCAATAAAT *isiB*
AAAAGTATTCTCAAGATAT *znuA*
AGAAATATCCTGATTATAT *psaK*
ATTAAAAACCACCAAAAAT *hutA2*
ATATAAATAGTCAATAAGT *all1127*
AAAAAAATACTCAATATGT *all1804*
AAAAATATACTGAAATTGC *all3903*
AATTAAATTCAGAATTAAT *alr4585*
ATTAAATTTCTGATTAAAT *all4246*
CTAAATAATCTCATTTAGT *alr0190*

Figure S2. The *Anabaena* sp. PCC 7120 FurA regulatory consensus sequence. (**A**) Sequence homology of the *Anabaena* 19-bp FurA box consensus with Fur binding consensus sequences from other eubacteria. (**B**) Proposed model for the FurA-DNA interaction, representing that at least two FurA monomers (shown as ovals) bind inverted heptamers (shown as arrows) within the 19-bp consensus. The model 7-1-7 of interaction to several naturally occurring FurA boxes are also indicated.
